# Factors that Influence Intake to One Municipal Animal Control Facility in Florida: A Qualitative Study

**DOI:** 10.3390/ani7070048

**Published:** 2017-06-30

**Authors:** Terry Spencer, Linda Behar-Horenstein, Joe Aufmuth, Nancy Hardt, Jennifer W. Applebaum, Amber Emanuel, Natalie Isaza

**Affiliations:** 1 College of Medicine, University of Florida, Gainesville, FL 32611, USA; nhardt@gmail.com; 2 Colleges of Dentistry, Education, Veterinary Medicine, & Pharmacy, University of Florida, Gainesville, FL 32611, USA; Lsbhoren@ufl.edu; 3 George A. Smathers Libraries, University of Florida, Gainesville, FL 32611, USA; mapper@uflib.ufl.edu; 4 College of Liberal Arts and Sciences, University of Florida, Gainesville, FL 32611, USA; jennyapplebaum@ufl.edu; 5 College of Health & Human Performance, University of Florida, Gainesville, FL 32611, USA; amberemanuel@ufl.edu; 6 College of Veterinary Medicine, University of Florida, Gainesville, FL 32611, USA; isazan@ufl.edu

**Keywords:** animal shelters, GIS mapping, socioeconomic disparities, grounded theory, pet abandonment

## Abstract

**Simple Summary:**

Animal shelters try to save homeless dogs and cats by returning lost pets to missing owners, adopting animals to new homes, and by reducing intake. We mapped the annual intake of one county animal shelter to discover where the homeless animals came from and selected one area of high-intake for stray adult dogs to study. We performed field interviews and reviewed available census and child-maltreatment data to create a theory about why so many stray dogs came from this study area. The study-area residents experience multiple socioeconomic challenges secondary to poverty including: interpersonal violence; housing instability; and lack of access to reliable transportation and communication services. Such factors lead residents to view domestic dogs not only as pets, but also as commodities that can add income to households, and often as burdens that results in pet abandonment. The community-specific data collected in this study can drive creation of strategic solutions for preventing pet abandonment and serve to reduce intake of stray dogs to the local animal shelter.

**Abstract:**

This qualitative study identified a study area by visualizing one year of animal intake from a municipal animal shelter on geographic information systems (GIS) maps to select an area of high stray-dog intake to investigate. Researchers conducted semi-structured interviews with residents of the selected study area to elucidate why there were high numbers of stray dogs coming from this location. Using grounded theory, three themes emerged from the interviews: concerns, attitudes, and disparities. The residents expressed concerns about animal welfare, personal safety, money, and health. They held various attitudes toward domestic animals in the community, including viewing them as pets, pests, or useful commodities (products). Residents expressed acceptance as well as some anger and fear about the situation in their community. Interviewees revealed they faced multiple socioeconomic disparities related to poverty. Pet abandonment can result when pet owners must prioritize human needs over animal needs, leading to increased shelter intake of stray dogs. Community-specific strategies for reducing local animal shelter intake should address the issue of pet abandonment by simultaneously targeting veterinary needs of animals, socioeconomic needs of residents, and respecting attitude differences between residents and shelter professionals.

## 1. Introduction

Mapping data with the aid of geographic information systems (GIS) is commonly-used to visualize relationships and analyze trends in industries such as, government, healthcare administration, and emergency management [1]. Animal shelter research has previously used this technology to characterize pet-adoption patterns and intake of dogs and cats [2,3,4,5]. In addition, GIS-mapping technology was previously used in Alachua County, Florida to: (a) identify neighborhoods with the greatest health disparities; (b) advocate for community outreach services; and (c) develop a mobile clinic and the Southwest Advocacy Group (SWAG) family resource center to deliver primary healthcare as a result [6].

In a survey of pet owners desiring to surrender their dogs to an animal shelter from a zip code associated with lower income addresses, researchers found that cost of veterinary care was a primary factor in their decision. This study also revealed secondary factors that influenced pet surrender, including: income, landlord issues, behavior issues, moving, lack of time, and new children [7]. In another study researchers surveyed pet-owners surrendering their large-breed dogs to identify strategies for keeping these harder-to-adopt dogs out of shelters. The study yielded no universally applicable intake-diversion strategies. This led the authors to conclude that community-specific solutions were needed to deter intake of large-breed dogs surrendered to animal shelters [8]. 

However, in addition to being surrendered by their owners, dogs enter municipal shelters impounded by animal control as homeless strays or as legal impounds during humane investigations or rabies quarantines. In fact, intake of strays typically represents the greatest volume of pets that enter municipal animal shelters [9]. No published studies to date have investigated community-specific factors that influence intake of stray dogs to an animal shelter. 

The purpose of this study was to use qualitative-research methods to explore a community from which high numbers of stray dogs entered a local municipal animal shelter. The community was selected by visualizing annual shelter intake data on GIS maps. Researchers would then interview a sample of residents and explore community-specific factors that might influence the shelter’s intake of stray dogs from the selected study area. The animal shelter selected for this project, operated by the Alachua County government, functions as animal control and is the only open-admission shelter in the county that accepts owner surrenders, strays, and legal impounds. There is one other limited-admission humane society in the county that transfers pets from this municipal shelter and accepts owner surrenders on a space-limited basis. 

## 2. Materials and Methods 

Research activities took place between January 2015 and January 2017. Investigators first worked with staff of the local animal shelter to improve the accuracy of their intake data and entry systems. Intake data reports were collected from the shelter’s Chameleon Software (HLP, Inc., Littleton, CO, USA) between August 2015 and July 2016. Researchers standardized, or cleaned, intake addresses in order to match addresses to latitude and longitude geographic coordinates, a process called geo-coding. A GIS was used to spatially visualize the data. The geo-coded intake data was then aggregated to a uniform grid and overlaid with analyzed historical child maltreatment data to visually aid in study area selection. Researchers did not perform further spatial analysis of the data.

One study area was selected to investigate for this project. Multiple sources of information were used to explore the study area, including: GIS maps, public records, census data, field observations, and semi-structured interviews with residents of neighborhoods of interest [10]. Researchers analyzed the compiled data by applying a constructivist, grounded-theory (GT) approach as described by Charmaz [11] to identify any unique themes that arose from the collected data. 

### 2.1. Animal Intake Data Collection, Cleaning, and Geo-Mapping

A total of 3747 intake records were collected throughout the one year study period, of which 846 records were eliminated through the data cleaning and geocoding process, leaving 2901 clean records. The cleaning process removed incomplete addresses, addresses outside of the county, and addresses related to veterinary care facilities or government related buildings. Addresses not located during the geocoding process were also removed. The clean data percentage of the final retained geo-coded data available to map was 77.42%. Lastly, the 2901 records were aggregated to 1608 unique addresses and summarized by species categories. Environmental Science and Research Institute’s (ESRI) Geocoder accessed through ArcGIS Online was used to geocode the addresses [12]. The geo-coded coordinates were then spatially visualized using ESRI’s ArcGIS ArcMap program release 10.5 [12]. A county wide half-mile square polygon grid, or fishnet, data layer was created to further aggregate geo-coded intake data to density per half-mile and thus facilitate uniform comparisons of the distribution and areal density of the spatial intake data. Addresses of veterinary care and governmental offices were geocoded to create an animal resources spatial layer, which included the Alachua County shelter. Additional spatial data layers consisting of the US Census’s socio-economic American Community Survey (ACS) Census 2011–2015 block groups, a county boundary, roadways, lakes, and rivers were acquired from the Florida Geographic Data Library [13]. Previously analyzed child maltreatment areal density data was provided courtesy of one co-author (NH) [6]. To standardize visual comparisons the maltreatment density data was resampled to the half-mile square fishnet grid. All spatial data layers were projected to a common planar coordinate system, the Florida Modified Albers (FMA) projection coordinate system. Animal intake density and child maltreatment density spatial data were simultaneously visualized for comparisons. A study area that contained high areal densities per half-mile common to both data sets was selected. The study area boundary GIS layer was created by selecting the 5 contiguous U.S. census block groups that contained the identified overlap. The census block group boundaries were then dissolved using ArcGIS to create a single study area boundary. Socio-economic summary statistics for the study area were generated.

### 2.2. Field Observations 

The field-research team consisted of three graduate students (Jennifer W. Applebaum, Dorothy Berry, and Britan Ethridge) and three faculty members (Terry Spencer, Linda Behar-Horenstein, and Amber Emanuel) who in advance of field work practiced to develop consistency. Researchers conducted multiple site visits looking for: living conditions of pets and people, challenges to pet ownership, availability of veterinary resources, personal safety concerns, language barriers, and best places to post recruitment flyers as well as residents to be interviewed (See Appendix A: Field Observation Guide).

### 2.3. Semi-Structured Interviews

Researchers interviewed a convenience sample of 39 volunteers recruited from within the study zone by use of posted flyers, canvassing, and tabling at community-gatherings. Each interviewer followed a semi-structured interview guide (See Appendix B: Interview Guide). Interviews were recorded, transcribed by a professional transcription service, and then entered into NVivo 11 software (QSR International Pty Ltd, Doncaster, Australia) for open-coding. Each volunteer who completed the interview received compensation of a $10 VISA card. To ensure anonymity, interviewees created unique personal codes that consisted of the initials for their parents and digits for their birth date. Interview questions were designed to gather information about family demographics and other socioeconomic-risk factors that might affect the welfare of dogs and cats in the study area, such as: mental and physical health, housing insecurity, interpersonal violence, and communication disparities. The questions were also intended to elicit attitudes toward domestic animals and ideas about the causes and solutions for the issue of animals entering the county shelter from the study area. Participants were White (22%), Black (53%), Hispanic and Other/Mixed Race, both (8%), 14% were retired, 33% unemployed, 23.5% employed, 23.5 % disabled, and 6% did not report this information.Twentynine percent had less than a high school education, 34% held a high school diploma or equivalent and 34% had some college or higher education. The remaining 6% did not report this information.

### 2.4. Analysis of Interview Transcripts

Two researchers (Terry Spencer and Linda Behar-Horenstein) applied grounded theory (GT) analytical techniques to inductively analyze the transcribed interviews in order to better understand the interviewee’s perspectives, explain the phenomenon of interest in the interviewee’s words, and to provide a framework for further study. They independently open-coded the data line-by-line in the transcripts then met to reach consensus about the initial coding and to agree on emergent themes [14,15]. The GT approach ensured that the researchers developed a deep understanding of the data by: looking and listening for cues about feeling and meaning; looking for how, when, and why people act; looking for what people do as well as what they say; and taking a critical stance toward the data, rather than the participants. Direct quotes were extracted from the coded-data to support the themes. This rigorous and systematic approach allowed the researchers to feel confident that what they report is representative of participants’ perspectives.

## 3. Results

### 3.1. Animal Intake 

The achieved cleaned, geocoded address result of 77.42% is 2.58% less than the ASPCA’s recommended 80% cleaned data rate [16]. Intake mostly consisted of stray adult animals. Alachua County took in slightly more cats than dogs. Animals less than 6 months of age were classified as juveniles, but not all intake data included an age estimate. We did not use intake data that recorded the reproductive status of the animals because that data was not confirmed by a shelter veterinarian and the status sometimes changed during the animal’s stay in the shelter (from not sterilized to sterilized) (See Appendix C).

### 3.2. GIS-Maps

**Figure 1:**
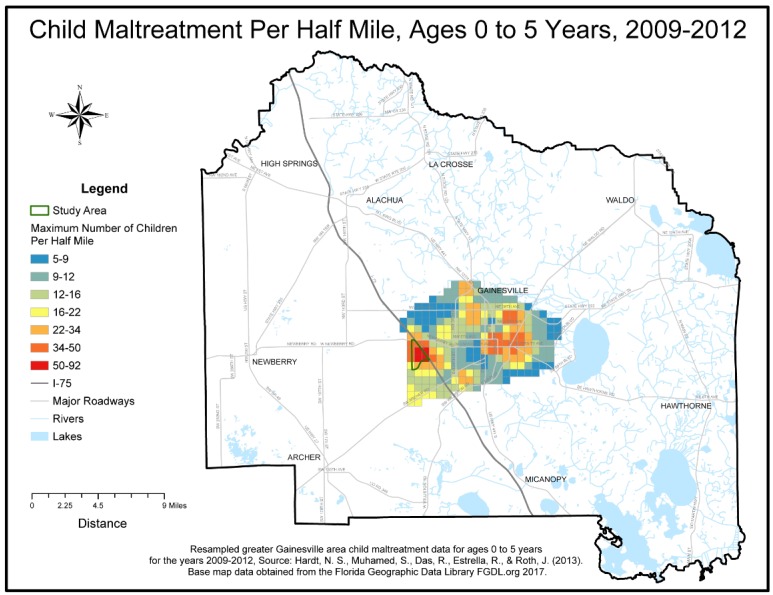
Child Maltreatment Density Map.

**Figure 2:**
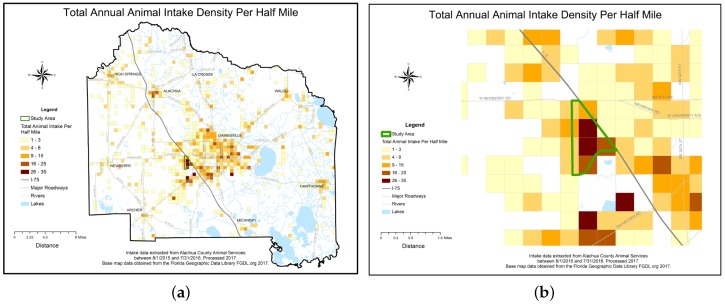
(**a**): Total Annual Animal Intake Density Map. (**b**): Study Area Close Up.

**Figure 3:**
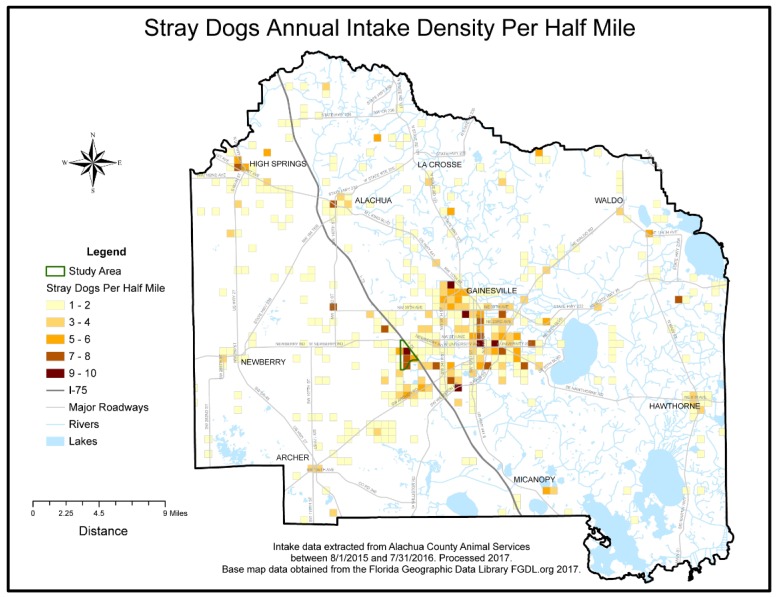
Stray Dog Annual Intake Density Map.

**Figure 4:**
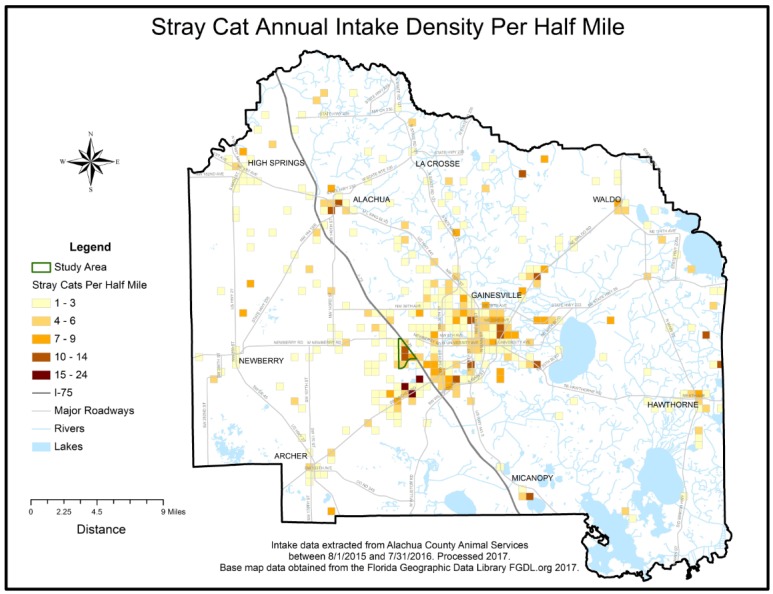
Stray Cat Annual Intake Density Map.

**Figure 5:**
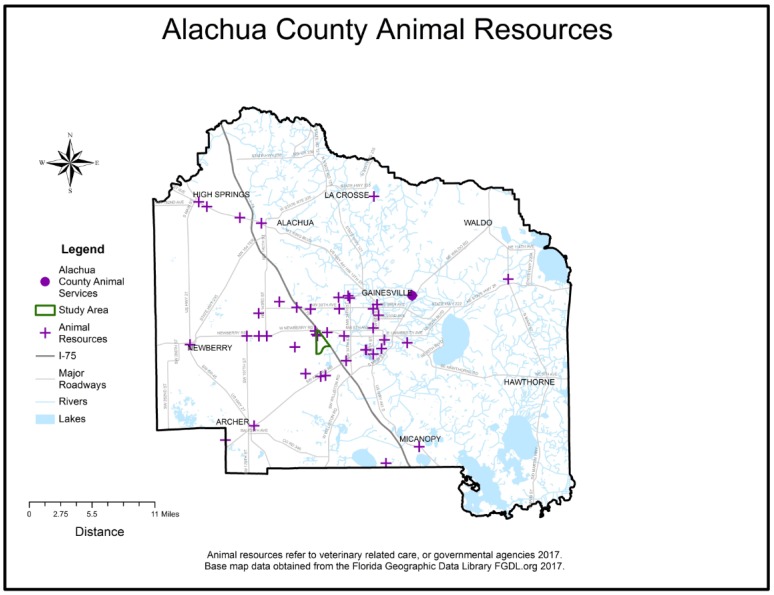
Alachua County Veterinary and Governmental Animal Resources.

Visualization of the clean, geocoded intake data revealed an area of high intake contained in 5 contiguous census block groups in Alachua County, Florida, which visually overlapped with a high-density of previously mapped Alachua County child maltreatment cases [6]. The outline of the 5 contiguous census block groups was used to determine the study area boundary and served as the focus for field investigations. Study area shelter records were aggregated to 59 unique study area addresses. The number of animals at each address ranged from minimum of 1 to a maximum of 8 per address. The average number of animals was 2.1 per address and the standard deviation was 1.8. The species maps revealed that a cluster of high animal-intake, particularly for dogs, overlapped with the child maltreatment data within the boundaries of the study area. A cluster of high-intake for cats occurred slightly south of the boundaries. (See Figure 1, Figure 2, Figure 3, Figure 4 and Figure 5). The Alachua County shelter location in relation to the study area can be seen in Figure 5. The distance from the study area center to Alachua County animal Services location is approximately 19 km by road and approximately 14 km by straight line distance.

### 3.3. Field Observations

Pet policies varied widely among the interviewees because each apartment was privately owned by a landlord who set policy rather than a single property management group. Multiple veterinary clinics and a pet-friendly county park were near to the study area. A non-profit community resource center (SWAG) opened in 2012, was centered in the area and offered medical and dental services, a food bank, a community garden, computers, and a children’s play area. County bus service was available, but ran infrequently on the weekends. The majority of bus stops were not protected from the weather. Pets were not allowed on public transportation unless they fit in carriers. 

**Figure 6:**
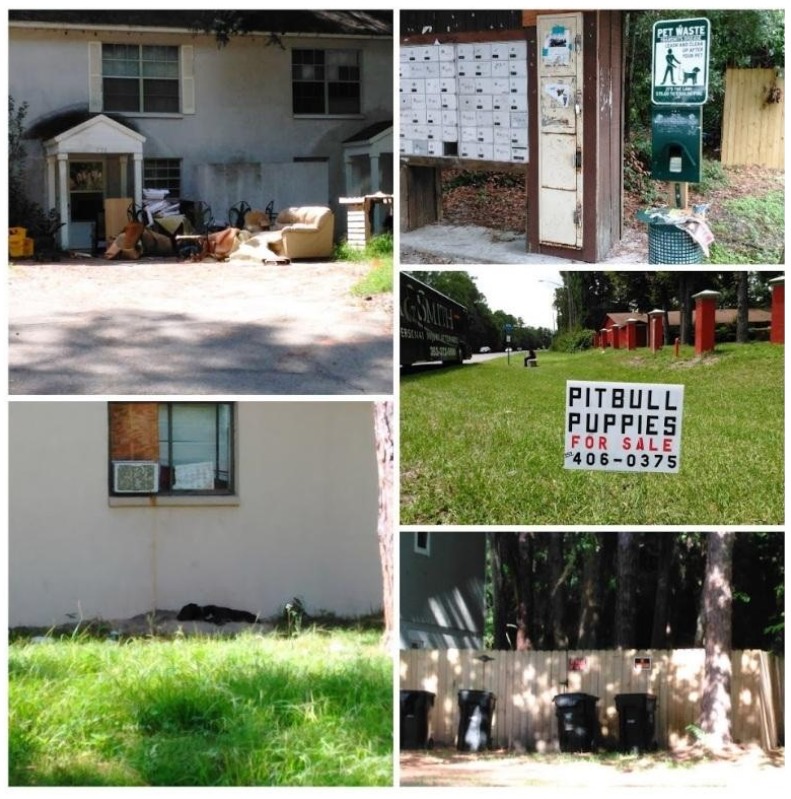
Images from Field Observations.

Pet dogs were seen walking on leashes, off-leash under voice control, and tethered. One free-roaming dog and several free-roaming cats without visible collars or ear-tips were seen. The presence of pet-waste receptacles suggested that this was a pet-friendly neighborhood. Animal Control Officers reported concerns about crime in the study area, as well as ongoing issues with improper identification and confinement of dogs in the neighborhoods. One case of dog-fighting had previously been investigated in the study area. Many residences displayed signs stating, “No Trespassing” and “Beware of Dogs,” which served as visual evidence of personal safety concerns present in the study area. Signs advertising pit-bull puppies for sale were also prominent. Evidence of recent evictions alluded to housing instability (See Figure 6).

### 3.4. Community and Participant Demographics

Block group census data was extracted from the 2011–2015 American Community Survey for the area of interest for this study, summed, and averaged (mean). The study area consists of 823 acres and a total population of 7921 people living in 3130 households. The household poverty level of the interviewees was determined by comparing reported family size to the 2017 federal poverty guidelines for the 48 contiguous states and the District of Columbia (See Table 1).

### 3.5. Interview Findings

Only 35 of the 39 interviews were analyzed because one interview was interrupted before it could be completed and three recordings were not audible for transcription. 

Fewer than half of the interviewees, 16 (46%) reported keeping pets in their homes. Seven were dog only homes (three kept single dogs, four kept two dogs). Five were cat only homes for one to three cats. Four homes kept both cats and dogs; including one with a single cat and single dog, two with two cats and one dog, and one home with one cat and two dogs. Pet dogs were described as either mixes or purebreds of various sizes. Cats were described as either indoor-only (one was walked outdoors on a leash) or allowed to freely roam outdoors. Nineteen (56%) of the interviewees reported not keeping pets in their homes. However, because they reported interacting with community and neighborhood pets, understanding the scope of their experiences with animals was considered important.

Many 22 (62.9%) interviewees mentioned community cats (unowned, free-roaming cats), although only 3 (8.6%) felt these cats were a nuisance. CAZ09 said “They don’t seem abused or nothing” when describing the cats in the neighborhood “laying around that need somewhere to go.” CEK16 said, “You see more cats than stray dogs” in the neighborhood. Several mentioned feeding the community cats, such as CLL12 who said, “I know it’s a lot of cats on my street. Mostly the people that stay on my street, it’s at least 3 or 4 houses that they all go to and they feed them. Like 8 or 10 cats.”

The thirty-five interviewees reported frequent relocations within the past five years; 21 (60%) of respondents reported having moved from one to nine times within the past five years, and 11 (31.4%) of those who had recently moved reported currently keeping pets in their homes. We did not determine whether the pets had moved with the residents or had been obtained after the most recent move. 

Some of the thirty-five respondents revealed previously experiencing controlling or threatening behavior by their partners toward themselves and their pets. Thirteen (37.1%) of the interviewees reported that they had previously felt controlled by their partners; 7 (20%) reported feeling afraid of their partners; 11 (31.4%) said they had been threatened by their partners; and 3 (8.6%) reported that their pets had been threatened, injured, or that fearing for their pet’s welfare influenced their decision whether to leave or stay with a partner.

Thirty-four interviewees responded to questions about their physical and mental health. Ten (29.4%) rated their physical and mental health toward the fair to poor end of the rating scale. Twelve (35.3%) considered their physical and emotional health to be good to excellent. The remaining 12 (35.3%) considered their health to be mixed on the rating scale. 

When directly asked about using dogs and cats as commercial commodities, (i.e., buying, selling, trading, breeding, betting, or gambling on animals), opinions varied. Most 23 (65.7%) disagreed with dog-fighting either because it was cruel or against the law. One interviewee (2.9%) felt it was all right to gamble on dogs, and a few 6 (17.1%) suspected illegal dog-fighting activities were occurring in their community but had not witnessed it. Some 10 (28.6%) thought it was all right to breed dogs and cats, 5 (14.3%) specifically agreed with selling puppies or kittens, and only 11 (31.4%) did not agree with breeding animals. Two (5.7%) suspected that dogs were being bred in the community to be used for dog fighting. Most 23 (75.7%) claimed they saw no evidence of breeding or selling of animals. MJM17 said “There’s a lot of buying and selling” in the neighborhoods and there were no signs posted because the transactions occurred though “word of mouth and internet.” CSN07 did not see a need for breeding and proffered that there are “so many that are in shelters already that they need to stop breeding their own.” He suggested if people wanted a dog that they find a rescue dog. JJT19 commented that he had seen signs that pit bull puppies were for sale at the entrance of the neighborhood. He remarked that if there were dogs needed to be adopted “especially in that breed that you probably don’t need to be breeding them.”

Three themes emerged from the coded interview data related to concerns, attitudes, and disparities. The interviewees expressed concerns about animal welfare, personal safety, health, and money. They expressed how they regarded dogs and cats as household pets but also as useful commodities that could provide protection or produce money through breeding litters or betting on dog fights. Some viewed stray dogs and cats as pests in the neighborhoods. Others were indifferent to the animals in the neighborhood or resolved to accept the conditions they witnessed in the community. The disparities discussed by interviewees were associated with poverty (i.e., evictions, disabilities, jobs loss, financial insecurity, limited access to transportation and communications, risks for personal safety) (See Appendix D: Themes and Example Codes).

The interviewees explained why they thought animals ended up in shelters. Surprisingly, the cost of caring for pets was mentioned by just 9 (25.7%) of the interviewees. Merely 3 (8.6%) mentioned pet deposits as a deterrent to pet keeping and 4 (11.4%) mentioned size or breed restrictions as a problem. However, 14 (40%) of the interviewees suggested that abandonment of pets was the main explanation for animal homelessness in their neighborhood: 13 (37.1%) said pet keepers were unable to provide proper care and 10 (28.6%) suggested uncontrolled reproduction was an issue. Very few 1 (2.9%) suggested the problem was due to distance from veterinary care or sterilization services. DJJ16 explained it was “because a lot of people don’t care for them or they move and can’t take them where they’re moving to.” MWA22 said, “I guess the people that move and just leave their animals behind.” SFF07 stated that, “They don't care about the dog no more.” JJT19 suggested problems emanated from the properties being primarily rental and the high incidence of evictions. Researchers regard abandonment of pets as the act of knowingly leaving an animal at a location where they will not receive minimum care, while no longer taking responsibility for the care of the animal. This differs from voluntarily surrendering an owned animal to the shelter where they will receive care.

The interviewees offered a variety of suggestions for keeping pets out of shelters. The most commonly proposed solution was for more sterilization of dogs and cats in the community 9 (25.7%) followed by confining pets 7 (20%) and taking proper care of the pets 6 (17.1%). This was best expressed by CEK16 who suggested, “Feed them, take care of them, and give them shots.” Other proposed solutions included educating residents about how to provide for the needs of pets 5 (14.3%), providing free veterinary care 4 (11.4%), and offering temporary shelter for pets 3 (8.5%). DAM18 suggested “temporary shelter for animals” was required when people experienced situational problems so that they could “get their animals back.” MAM12 described pet homelessness as endemic to the community culture, “You know this neighborhood has changed so much over the past years. There are just a lot of irresponsible people in many ways that live here.” CEK16 commented on the responsibilities associated with caring for pets, “Anybody who has a pet is going to become attached to the pet and they want to take care of their pet. And if they are not able to take care of the pet they have to give it up.”

## 4. Discussion

This qualitative study described that poverty-related disparities increased the risk for pet abandonment within a geo-coded study area from which high numbers of stray dogs entered the local animal shelter. Field interviews with residents revealed their concerns about animal welfare, money, personal safety, and health. The respondents also revealed that their attitudes toward domestic animals varied from useful commodities (products) that could supplement income or guard property, to pests, or as household pets. This finding supports previous studies of attitudes toward animals as pets, pests, or profit [17]. Community-specific strategies for reducing local shelter intake of stray dogs should target the issue of pet abandonment by co-addressing veterinary needs of animals, socioeconomic disparities of residents, and attitudinal differences between residents and shelter professionals. 

**Limitations:** Because more residents were available to interview when the SWAG Family Resource Center was open, the research team interviewed a more unemployed and older set of community residents who were using the services of the Resource Center than the census mean predicted. This convenience sample could have skewed the study findings. In addition, interviewees were informed at the time of recruitment that they would receive a $10 gift card in exchange for their time, which might have influenced the study sample. However, recruitment flyers posted in the study area advertised the $10 gift card, but did not yield any volunteers to be interviewed. The interview findings may only be representative of those community members who match the demographics of the residents we sampled. Thus findings of this study may not be generalizable to the larger community. 

Our investigation revealed that residents were concerned about the welfare of homeless animals in their neighborhoods. Several previous studies have documented a decrease in animal homelessness after increasing access to spay-neuter surgeries within a city or a postal zip code [18,19,20,21]. Indeed, a nationwide emphasis on sterilization of pets has resulted in a corresponding decrease of animal intake to shelters in the United States over the past few decades to an estimated low of 7 million animals in 2015 from a high of 13 million in 1973 [20]. The current study area is located within a well-resourced veterinary community. Residents have access to nearby private veterinary clinics, veterinary college, veterinary clinic serving low-income clients, as well as a low-cost sterilization clinic for pets farther away on the northeast side of town. This community is definitely not a “veterinary desert” as previously documented by GIS mapping in Atlanta, GA, USA [22].

Despite ready access to veterinary services, our investigation revealed that few puppies, kittens, or community cats from this study area have benefitted from veterinary care. In addition to personal concerns about money, disparities with communications, transportation, and health might explain why some of the study-area residents failed to sterilize their pets. With intermittent phone and internet access, it can be difficult to schedule a veterinary appointment. Limited bus schedules and pet restrictions on public transportation likely present additional obstacles for accessing veterinary resources when they are not located within easy walking distance. Personal-health challenges might further limit a pet-owner’s ability to obtain veterinary services without additional assistance. One solution for overcoming such disparities might be to regularly offer veterinary services, in addition to medical and dental services, at the SWAG community resource center located within the study area. Applying such a “one health” approach to the social determinants of community health could greatly improve animal welfare in this neighborhood.

The problem of abandoning unwanted pets, rather than surrendering them to the safety net of a local animal shelter, was identified during this investigation. This issue could be exacerbated by the number of socioeconomic disparities faced by community residents rather than by a lack of bonding with pets. In fact, the majority of interviewees responded favorably toward community cats and dogs rather than regarding them as pests. Unfortunately, the municipal animal shelter in this study charges a fee to surrender a pet and an additional fee to transport an unwanted pet to the county shelter. Such fees likely deter pet owners of this neighborhood from “doing the right thing” when faced with multiple and pressing socioeconomic challenges. Therefore, it might seem easier to turn a pet loose in the neighborhood where another neighbor might choose to care for it than to transport that unwanted pet across the county to the animal shelter during specific hours of operation and also pay a fee. A simpler solution would be to waive owner surrender and transport fees from this neighborhood.

Surprisingly, GIS maps of increased stray dog intake from this neighborhood visually corresponded with previously made GIS maps of increased cases of child maltreatment from the same study area. This potential correlation between homeless animals and child maltreatment hinted at a community facing multiple challenges and prompted the research team to further investigate the neighborhood. Certainly this observation deserves additional study to assess any relationship between adverse childhood events and animal homelessness. In the meantime, this community might benefit from encouraging animal control officers to work jointly with local social services personnel to support both people and their pets. For example, the municipal animal shelter could use volunteers to repair fences in order to better confine dogs in their owner’s yards. The county could also provide pet-friendly emergency sheltering for families experiencing episodes of domestic violence or eviction. Such interagency cooperation might encourage pet retention rather than pet abandonment. 

However, such solutions will likely not change the attitudes of those residents who view animals as commodities for protecting personal property and increasing personal income rather than as pets. This study reveals the need for a different solution that addresses the local economy of pets in order to reduce the urge to breed, sell, and gamble on dogs. 

In 2011, the ASPCA collaborated with Portland, Oregon to provide free and reduced-cost veterinary services for geo-coded neighborhoods associated with high-intake to the local animal shelter and then assessed whether the intervention decreased shelter animal intake. This study documented a small reduction in the intake of surrendered cats but not a significant decrease in intake for surrendered dogs or any strays [23]. Perhaps these limited results were due to a mismatch between the proffered solutions and the true problems experienced by the residents of the study area. Our study indicates that community-specific findings, such as attitudinal differences and disparate socioeconomic conditions, should be addressed when designing strategies to reduce stray dog intake to the local animal shelter. 

The institutional culture of animal shelters influences how the animal welfare community defines social problems, poses solutions, and serves its clients [24]. Increasing access to free or low-cost veterinary services is the primary solution offered by animal shelter professionals for solving the problem of unwanted domestic pets. However, as this study shows, not all who live with dogs and cats view these animals as pets nor is cost of pet care their primary concern. It is possible that animal welfare professionals are overlooking other solutions to the problem of pet homelessness because they lack familiarity with the lived-experiences of community residents or they apply a “moral certainty” to saving animals that divides clients into those who are “good” and “bad” pet caretakers [25]. For example, shelter professionals might view pet owners who choose to breed their pets as a “bad” owners, even if the money obtained by selling the litters helps the families put food on their tables. As poverty increases, shelter and security concerns arise for families, imperiling their ability to care for pets. As the urgency of socioeconomic disparities increases, pets become burdensome to at-risk families, which can result in their abandonment in an effort to preserve family resources. 

## 5. Conclusions

In order to reduce the intake of homeless pets, shelter professionals should attempt to connect and empathize with their clients rather than judging them and using “institutional thinking” to solve problems. Giving a dog free vaccine, an identification tag, and a low-cost sterilization might be a solution to a veterinary-related problem, but it isn’t the solution needed by a pet living in a household facing a socioeconomic issue such as eviction, domestic violence, or a family medical emergency. As this and previous studies allude, community-specific solutions are necessary to solve community-specific problems that involve both humans and animals. Although this study was specific to one neighborhood served by a municipal animal shelter in Florida, the findings are applicable to other shelters and communities. Talking directly with residents of communities responsible for high-intake to animal shelters should help us discover what residents need, so we can develop targeted solutions for addressing animal welfare issues.

## Figures and Tables

**Table 1:** Comparison of Demographics for Census Block Group and Interviewees.

Demographics	Census Data	Interviewees
Average household size (people)	3.87	2.5
Household income:		
Below poverty line	34%	80%
Above poverty line	66%	3%
Unknown	-	17%
Gender:		
Male	47%	50%
Female	53%	50%
Education:		
Less than high school	9%	29%
High school diploma or equivalent	29%	34%
Some college or higher ed	62%	34%
Race and ethnicity:		
White	48%	22%
Black	45%	53%
Hispanic	1%	8%
Other/Mixed race	7%	8%

## Appendix A. Observation Field Notes Guide

***Part 1: Initial Observations of GIS-Mapped Neighborhoods***

1. Schedule an initial one-hour ride-along with an animal control officer or community service officer who is familiar with each GIS-mapped neighborhood to be observed.
2. Prior to the initial observation session, prepare to compile **FIELD NOTES** for each neighborhood that will include:
  - the day of the week and date of the initial observations
  - the scheduled start and stop times of the initial observations
  - descriptive name for each observed neighborhood
  - the boundaries for each neighborhood to be observed
  - who will be present for the initial observations, including your name as the observer
  - Background information from the accompanying animal control officer or community service officer that describes their familiarity with residents and domestic animals of each neighborhood (What is their professional knowledge about each neighborhood? What are the common types of service calls they make in each neighborhood? Do they know of any particular challenges or issues faced by the residents or dogs and cats of the neighborhoods? Why do they think so many dogs and cats enter the county animal shelter from these locations? What do they think is needed to prevent dogs and cats from these neighborhoods ending up at the county animal shelter?)
3. During the initial observation session in each neighborhood, and with the ride-along officer’s assistance, record or document in the FIELD NOTES evidence of/that:
  - dogs and cats being kept as household or personal **pets**
  - the neighborhood is **pet-friendly**
  - dogs and cats being cared for as community animals or **loosely-owned** animals
  - dogs and cats are viewed as **pests** or problematic in the community
  - dogs and cats are viewed as **products** in the community (for breeding, gambling, fighting, selling, trading, etc.)
  - specific neighborhood **culture or language** exists
  - specific neighborhood **socioeconomic status** exists
  - access to basic **community services** for people
  - access to pet care and **veterinary care services**
  - residents of the neighborhood feel **safe**
  - special **challenges/issues** facing this community
  The evidence you collect should note or document the presence or absence of such things as (sort of like a scavenger hunt):
  - Freely-roaming animals
  - Pets on leashes
  - Pet collars (and note types of collars, such as heavy chains, pinch, harness, etc.)
  - Tethered pets
  - Pet-feeding stations
  - Pet-water bowls
  - Pet-food bowls
  - Pet housing
  - Fenced yards
  - Dog parks
  - Animals feeding from waste containers
  - Lost pet flyers
  - Posted signs restricting animals
  - Advertisements for "pet-friendly" housing or businesses
  - Posted policies restricting breeds of dogs
  - Public transportation
  - Community centers
  - Libraries
  - Schools
  - Churches
  - Grocery stores
  - Recreation areas
  - Veterinary care centers
  - Foreclosures
  - Real estate for sale or for rent
  - Whether people are active outdoors and estimate their ages
  - Language used on posted signs
  - Predominant housing type (apts/mobiles/single family/tents/dorms) and density of residences.
  - Whether housing has bars on windows and doors
  - “Community watch” or community protest signs
  - Predominant types of businesses in neighborhood
  - Other pertinent items, such as abandoned houses, construction, etc.
4. After each observation session, take time to reflect on everything you observed or documented. What information do you still need? What questions remain? What concerns you about the relationships between people and pets in this neighborhood? Add a summary of your impressions of each neighborhood to the Field Notes.
5. Compile the Field Notes, your summary, and debrief with the research team.

***Part 2: Follow-up Observations of GIS-Mapped Neighborhoods***

1. Schedule follow-up observation sessions to each neighborhood with a partner who can drive while you compile Field Notes. The purpose of the follow-up sessions is to validate your initial observations on different days of the week and at different times of day.
  - Plan to make a total of 4 observations in each neighborhood, including the initial observation session, which occurs on a: weekday during normal business hours; weekday evening; weekend day; and weekend evening.
  - Plan any additional observations only if necessary to answer questions that arise during the sessions or to clarify data you were unsure of from previous sessions.
  - Use the same format for your Field Notes as during the initial observation. Your follow-up sessions should each be one-hour in duration and serve to supplement the data you collect from prior observations.
  - Reflect and write your summary notes after each session.
2. Compile the Field Notes, your summaries, and debrief with the research team.

## Appendix B. Interview Guide

***PART I: Obtain Informed Consent and Create Personal ID Code***

A. Read page one of the *Informed Consent Document* to each volunteer, or ask him/her to read page one of the *Informed Consent Document*.
B. Have volunteer sign, date, and provide their mailing address on page two of the *Informed Consent Document* if they agree to participate.
C. Collect page two of the *Informed Consent Document* and give page one to the volunteer.
D. Ask the volunteer to create a personal identification code and remind them to note that code on their copy of the *Informed Consent Document* so they will have it in the future if they wish later to withdraw from the study. Volunteers create a person ID code using the following system:
  - First letter of mother’s first name
  - First letter of father’s first name
  - Middle initial of volunteer
  - Day of their birth (two digits)

EXAMPLE: my mother’s name is Rosie and my father’s name is Milton. My middle initial is G for Gale. I was born on the second day of the month. My personal code is: RMG02.

***PART II: Demographics (closed-ended questions)***

Inform volunteer that you will be recording the interview. Turn on the recorder and test that it is recording properly. Then begin the interview by asking the following questions:

Q1. What is your personal ID code for participating in this interview?
Q2. Please describe the location of this neighborhood and tell me whether you live or work here. *(EXAMPLE: This is Haile Plantation. I work here.)*
Q3. If you live here, please describe the type of housing you live in: *(EXAMPLE: is it an apartment, mobile home, condo, tent, dorm, assisted living facility, single-family home,* etc.)
  ***NOTE: if they do not live in this neighborhood, skip to Q4.***
  Q3A. How many people share your residence? *(CHOOSE ONE: I live alone; I live with _______others)*
  Q3B. Do any dogs or cats share your residence? *(CHOOSE ONE: No, Yes ________ dogs and _______cats)*
  Q3C. How many times have you moved (relocated your residence) in the past 5 years? *(CHOOSE ONE: 0 times, 1–2 times, 3–4 times, 4–5 times, more than 5 times)*
Q4. If you work here, please describe what you do: (*EXAMPLE: shopkeeper, clerk, law-enforcement officer,* etc.)
  Q4A. Do any dogs or cats live at your place of work? *(CHOOSE ONE: No, Yes _______ dogs and _______ cats.)*
Q5. How long have you lived or worked in this neighborhood? *(CHOOSE ONE: Less than1 year, 1–5 years, 6–10 years, 11 or more years)*
Q6. What is your sex? *(CHOOSE ONE: Male, Female, Other, or I prefer not to say.)*
Q7. What is your age? *(CHOOSE ONE: 18–25 years, 26–30 years, 31–40 years, 41–50 years, 51–60 years, 61–70 years, 71–80 years, 81–90 years, 91 years or older)*
Q8. What is the highest level of education you completed? *(CHOOSE ONE: I did not finish high school, GED or high school diploma, vocational certificate or some college, undergraduate degree, graduate degree (such as a master’s or PhD), professional degree (such as an MD or DVM), post-doc)*
Q9. How do you prefer to describe your racial and/or ethnic group? *(EXAMPLES: Hispanic White, Non-Hispanic Black, Asian,* etc.*)*
Q10. What is your primary spoken or written language? *(EXAMPLES: American English, Spanish, French, Chinese, Russian,* etc.*)*
Q11. What is the approximate annual income for you and your immediate family? *(CHOOSE ONE: less than $10K, $11–20K, $21–30K, $31–40K, $41–50K, $51–60K, $61–70K, $71–80K, $81–90K, $91–100K, More than $100K.)*
  Q11A. How many people are dependent on this income? *(CHOOSE ONE: only me, 2, 3, 4, 5, 6, 7, 8, 9, 10,* etc.*)*
Q12. How often do you have access to a computer and the internet for personal use? *(CHOOSE ONE: Always, Never, only Sometimes--if chooses Sometimes, ask for an explanation for why)*
Q13. How often do you have access to a smartphone (cell phone) for personal use? *(CHOOSE ONE: Always, Never, Sometimes—if chooses sometimes, ask for an explanation for why)*

***PART III: Health and Relationships (closed-ended questions)***

Q14. How do you rate your physical health? *(CHOOSE ONE: excellent, good, fair, poor)*
Q15. How do you rate your emotional health? *(CHOOSE ONE: excellent, good, fair, poor)*
Q16. Have you ever had a partner try to control you? *(CHOOSE ONE: yes, maybe, no)*
Q17. Have you ever had a partner of whom you were afraid? *(CHOOSE ONE: yes, maybe, no)*
Q18. Have you ever had a partner who threatened you? *(CHOOSE ONE: yes, maybe, no)*
Q19. Have you ever had a partner who threatened to hurt or kill your pet? *(CHOOSE ONE: yes, maybe, no)*
Q20. Have you ever had a partner who intentionally hurt or injured your pet? *(CHOOSE ONE: yes, maybe, no)*
Q21. Has concern about your pet’s welfare ever affected your decision-making about whether to stay or leave your partner? *(CHOOSE ONE: yes, maybe, no)*

***PART IV: Attitudes toward Animals, Attachment to Pets, Accessibility of Veterinary Resources (open-ended questions)***

Q22. Tell me about any dogs or cats that you keep or care for.
Q23. Tell me about any other dogs or cats that live within your neighborhood.
Q24. Tell me about any issues or problems you have with the dogs and cats in your neighborhood.
Q25. Tell me how you feel about breeding of dogs and/or cats in this neighborhood.
Q26. Tell me how you feel about betting (gambling) on dogs and/or cats in this neighborhood,
Q27. Tell me how you feel about buying/selling/trading of dogs and/or cats in this neighborhood.
Q28. How would you feel about a neighborhood dog or cat being euthanized (put down) at the county animal shelter?
Q29. Why do you think so many dogs and cats from this neighborhood end up at the county animal shelter?
Q30. What do you think would help keep dogs and cats from this neighborhood out of the county animal shelter?

***PART V: Confirmation of Findings***

Q31. Thank the volunteer for participating in this study and ask if you can contact them again to confirm the finding. (CHOOSE ONE: Yes or No)
  Q31a. If the answer is NO, this completes the interview.
  Q31b. If the answer is YES, ask them how to contact them when you are ready. NOT COLLECT THEIR NAME, you will refer to them using their personal ID code when you contact them—Record the phone number or other method they want you to use to contact them.)

## Appendix C. Annual Animal Intake for Alachua County, Florida

**Table:**

	**Count By Species and Age**			**Percent of 2901 Animals**		
Intake Age	Canine	Feline	total	Canine	Feline	total
Adult	1121	875	1996	38.64%	30.16%	68.80%
Juvenile	77	291	368	2.65%	10.03%	12.69%
Unknown Age	210	327	537	7.24%	11.27%	18.51%
	**Count By Species and Type**			**Percent of 2901 Animals**		
Intake Type	Canine	Feline	total	Canine	Feline	total
Stray	955	1279	2234	32.92%	44.09%	77.01%
Surrendered or Returned	409	211	620	14.10%	7.27%	21.37%
Confiscated	40	3	43	1.38%	0.10%	1.48%
Other Disposition	4	0	4	0.14%	0.00%	0.14%
Total	1408	1493	2901	48.53%	51.47%	100.00%

## Appendix D. Themes and Example Codes

**Table:**

**THEMES**	**Codes**	**Examples**
**CONCERNS**	**Animal Welfare**	**RCV12:** I think we can’t take care of them, don’t breed them. You see them [indiscernible] you see now these guys out here, under the sun, and I’m like it breaks my heart to see a dog like that. He sits there all day chained to a tree. **EEL05:** Sometimes they don’t have shelter at night. They are not getting fed on a regular basis. Just whatever they can scrounge. **MBE12:** I know of a lady trying to help sell little Chihuahuas. I drove them to Missouri and was bottle feeding this one all of the way over. How cruel, taking it away from its mother early age. To each its own, but—I’m powerless. **DAM18:** Cats are in their apartment and dogs are in their apartments. Until they put them out on a chain outside. Well, they do let the pit bulls outside. Well, they’re pit bulls, and when you pass by, they start barking. Chaining them out there—I heard they ate a few cats when they got off that chain.
	**Personal Safety**	**MWA22:** We ain’t in a very good neighborhood. **JJD12:** I’ve seen them [dogs in the neighborhood] but they’re not stray. Yeah they’re owned by others, yes ma’am. Yes, yes definitely [on leashes]. All but one. All but one they never have on a leash and I’m terrified of that dog. I’m just afraid of big dogs. I think it’s a pit. It’s like, um, like a pit bulldog. **CLL12:** I’m scared of anyone [dogs] that’s not mine. But for the most part I’ve never had a bad experience. **MJM17:** Because people don’t want to take care of their own animals. They just want to let them do whatever they want and that’s why dogs have to be—dogs and cats get put down because they want to attack people because they do what they want when they want to and when you try to change their habit you can’t. There are little kids around here.
	**Money**	**CLL12:** My neighbors have two dogs and it’s not the same situation. A small dog is like 6 months and really, really skinny. I try to feed sometimes but I can’t afford to feed it. **JJD30:** We did have to [pay] $200 [pet fee for cat in apartment]. That’s expensive for a cat. Like my husband back in the day, he used to work for (indiscernible). So [landlord] allowed us to pay him so much a month. We’re on limited income and have to pay the light bill and everything else, but he’s working with us. **OHM29:** The main problem when you have to pay the vet. The test is really expensive. I think maybe the community have to have options for veterinary care that’s free. Because one time I bring the pet to UF vet, they treat everything. It’s expensive, $2000. My son requested a loan from the college because he has scholarships. It doesn’t pay for [Indiscernible] he doesn’t want to lose the dog [Indiscernible] he wants to treat. It’s really expensive. Because my son is a student. I make eight hundred a month and we live alone.
	**Health**	**EJM18:** I would [have a cat] if I could. My daughter, she’s pregnant. The doctor told her (indiscernible). She can’t be around them. That’s the only reason I won’t bring the cat in. I wish I could open the door and bring them in. **MOE20:** It’s my daughter’s dog. My daughter, I told you she has got the heart transplant. They all fell in love with her. She fell in love with the golden doodle that used to come to the hospital visiting. So, they all got together with the breeder, and the breeder gave her the golden doodle, and throughout the—all the doodle owners, they all pay for everything for my daughter for the dog. They pay for the vet, groom, feed. They take care of it all. Once it gets to be a year old, it’s going to be put on as a service dog. They are paying for everything. It’s something for my daughter. But right now, it’s my dog. It bites too much right now. **DAM18:** I had two dogs and they took them to [low-cost vet clinic] and she took because I had hip replacement and she took one of my puppies away because I was sick and can’t take care of the puppy. And I still could have took care of the puppy. No. I wasn’t, [OK with that] because you know it was my dog and she kept convincing me that I—and I had to go to surgery. I don’t have any family except my boyfriend. It was hard—I should have took her back.
**ATTITUDES**	**Animals are pets**	**JJD30:** If you have got an animal, take care of it. Keep it inside. Like me, my cat, that’s my girl. I take care of her. She’s a member of my family. Everybody in the family loves her. If she wants to go out and use the bathroom, she will sit there. So, I keep my spray bottle because there’s a calico and a black and white one. The other day they literally tore her collar off. I just take a little water bottle and spray. I wouldn’t hurt nobody’s animal, but I’m going to protect mine at the same time. **FJL31:** I have one dog. Her name is Lee-Lou. That in Mandarin Cantonese means perfect. And she is. She was abused as a puppy. It’s actually my brother’s dog but he passed away a year ago and I promised him I would keep her forever. She’s 10 years older than I am and I’m 67 and she’ll healthy as a horse just like I am. We go walk about 6 to 8 times a day. I give her at least a mile to a mile and a quarter every day and I try to get 3 miles myself. **CAZ09:** I had a dog when I was like 11 or 12 and it got ran over. That really hurt me. [Indiscernible] because I get attached to them. I love them but I can’t get one because something happen to them that’s how I know other people feel about animals when they get hurt. I don’t get none of them.
	**Animals are commodities/products**	**CGR11:** I don’t think people should be able to breed. Certain people, especially if you want to know their background. Some of them be breeding dogs, having them fight. You know, I don’t know. They breed them, fight them. **MRJ14:** My neighbor has one, and he’s pretty good. Real friendly. And sometimes when they go out of town, we keep him. Pit bull but really trained and loves kids. He’s not no mean dog. He won’t bite unless you try to, you know, go outside, don’t know you, or you try to go in there or something like that. Yeah, really protective. More protection and part of the family. **CEK16:** Dogs do have litters. They can’t keep all of the litter so they sell the dogs. **DWM26:** But I was telling [Indiscernible] our daughter bets on dogs but I ain’t never seen it. **SRT02:** Yeah, it was a business. And were they purebred dogs like they have papers or—I don’t know that much. I know he has a website and that’s his main income in Gainesville. Yeah, I know him and some other guys that do dog shows and stuff like that. I know like some people have those stands or whatever to make dogs breed and stuff like that. But I don’t know. I never went inside and seen his operation.
	**Animals are pests**	**MJM17:** There’s a lot of dogs and cats around here but the thing about it is when people don’t want to have their dogs around no more they let them run loose. That’s a big deal because basically if you wanted a pet that’s your pet. Not other people’s. Other people trying to take care of your pets that you have that you let loose. A lot of them have owners but the owners let them run loose anytime they want until they want to come home. **MOE20:** The cats. They just roam. My cats never see outside. But other people, they’ve got the outside cats and they always run around in my yard. **JWG09:** Barking at 4 in the morning. **RCV12:** I just don’t like them hanging around because of thieves and stuff. Crapping in the yard.
	**Acceptance/Pragmatic**	**MWA22:** Just let the, the animal just stay around. They ain’t causing no trouble. Feed off the land. **SFF07:** If you’re doing it [breeding] for the right reason, yeah, but if you do it for the wrong reason, no. Right reasons to protect your family. If you got paperwork on the dogs and you’re doing it legal, yeah. It’s okay. **DWM26:** I would buy a dog. I would buy a pit dog. **MRJ14:** Because betting on it you get caught you’re going to jail. Fighters dog the same thing, going to jail. I’m against that.
	**Angry/Fearful**	**CLL12:** Hard to answer. I’m like an anti-person. [Indiscernible] I don’t know anyone so... **JCL21:** Because see I have to stay in my house. When I my house I get in my vehicle and I leave out of here. Come back home and go to my house again. I don’t really—I’m not really from this side of town. I was raised on the Southeast side of town. So that’s where all my family is so when I leave my house—Yeah. I don’t really know what’s going on out here. I have an American Rock and Pit mixed with Jack Russel. They bite. I keep them in my house. [Laughing] **JJD30:** Because me, I stay in the house. I go in, come out and get grandbabies and go to the grocery store. I stay by myself. **MRJ14:** Well, over there where I live, I don’t see that. Not where I live at. No. Well, I don’t go on that side. I just keep to myself.
**DISPARITIES**	**Economic insecurity**	**JFE21:** They can’t take care of them. Can’t take care of themselves. **MBE12:** Same way a lot of people end up over here. The poverty situation.
	**Housing insecurity**	**EJM18:** Yeah. It was the home in Bronson. We had the job situation. Someone stole my car, so I couldn’t get to work. So, the money wasn’t coming in, so they foreclosed and we had to move. We gave the cats to the shelter. **JJT19:** There is a lot of evictions around here. I own three units here. I don’t allow pets. Yes, we had one tenant who temporarily had his son’s pit bull, and the next thing we knew, there was a whole litter of puppies. They wrecked the whole back fence and chewed holes in the walls. **MOE20:** Well, what most of the new landlords are doing around here, they are making it pet free. No pits. There’s a pit bull right now—people are moving, and they have got to find a home for it. It’s a five-month-old puppy. They can’t find a place for it.
	**Transportation challenges**	**MBE12:** Now, I need a vehicle to put myself out of the neighborhood and change this. I have a driver’s license, which is—and I’m 63 though, finding a job, good luck. We just got buses on Sunday. What a God send. Thank you for the little blessing. They feel like they are throwing us a big bone. Look at the bus stops. If it rains, we are still standing in the rain. Go to any other neighborhoods and they have nice covered—don’t get me started. **OHM29:** Some people maybe don’t bring the pets in for the vaccine. [Indiscernible] I see some dogs. It’s really long trip, painful.
	**Communication challenges**	**MRJ14:** I don’t have neither one of them [smart phone or internet]. I have a government phone. Just plain phone. **SJR16:** Yeah, you got my cell number [to call back]. It’s disconnected, unless I pay.
	**Personal health challenges**	**DCA08:** No. I can’t [work]. I just got—I fell and broke my ribs. I just got out of rehab. That’s why I’m here **FJL31:** Unfortunately, I was born with congenital cataracts. I’m almost blind in my left eye because it’s hemorrhaged 5 times. **MRJ14:** I had three jobs. Not now. I’m disabled now.
